# Toll‐like receptors (TLRs): An old family of immune receptors with a new face in cancer pathogenesis

**DOI:** 10.1111/jcmm.16214

**Published:** 2020-12-18

**Authors:** Yazdan Mokhtari, Atieh Pourbagheri‐Sigaroodi, Parisa Zafari, Nader Bagheri, Seyed H. Ghaffari, Davood Bashash

**Affiliations:** ^1^ Department of Hematology and Blood Banking School of Allied Medical Sciences Shahid Beheshti University of Medical Sciences Tehran Iran; ^2^ Department of Immunology Faculty of Medicine Mazandaran University of Medical Sciences Sari Iran; ^3^ Student Research Committee Faculty of Medicine Mazandaran University of Medical Sciences Sari Iran; ^4^ Cellular and Molecular Research Center Basic Health Sciences Institute Shahrekord University of Medical Sciences Shahrekord Iran; ^5^ Hematology, Oncology and Stem Cell Transplantation Research Center Shariati Hospital School of Medicine Tehran University of Medical Sciences Tehran Iran

**Keywords:** cancer, immune system, inflammation, pattern‐recognition receptor, Toll‐like receptor (TLR)

## Abstract

In the dark path of tumorigenesis, the more carefully the cancer biology is studied, the more brilliant answers could be given to the countless questions about its orchestrating derivers. The identification of the correlation between Toll‐like receptors (TLRs) and different processes involved in carcinogenesis was one of the single points of blinding light highlighting the interconnection between the immune system and cancer. TLRs are a wide family of single‐pass membrane‐spanning receptors that have developed through the evolution to recognize the structurally conserved molecules derived from microorganisms or damaged cells. But this is not everything about these receptors as they could orchestrate several downstream signalling pathways leading to the formation or suppression of cancer cells. The present review is tempted to provide a concise schematic about the biology and the characters of TLRs and also summarize the major findings of the regulatory role of TLRs and their associated signalling in the pathogenesis of human cancers.

## 1 INTRODUCTION

Innate immunity not only represents the first line of defence against invading microbial pathogens but also is the first step towards the activation and stimulation of adaptive immunity. Upon exposure to bacteria, viruses, protozoa and fungi, innate immune cells including neutrophils, monocytes, macrophages, dendritic cells (DCs), natural killer (NK) cells and the complement system are activated. The response of the innate immunity to microbial pathogens relies on the specific host‐receptor detection of pathogen‐ and danger‐derived molecular signatures, known as PAMPs and DAMPs, respectively. When PAMPs and DAMPs are recognized by germline‐encoded pattern‐recognition receptors (PRRs), different types of cytokines would be released, which in turn attract secondary defensive immune cells. In the long list of PRRs, Toll‐like receptors (TLRs) are the most important ones. TLRs are one of the largest and most well‐studied families of PRRs, which were first recognized in the fruit fly, Drosophila melanogaster.^1^ Not only these receptors are one of the main components of innate immunity, infection diseases, and inflammatory conditions, but also they act as a bridge between innate and adaptive immunity. Apart from regulatory role in immune responses, TLRs have a hand in tissue homeostasis maintenance by regulating tissue repair and regeneration. But the mystery behind TLR functions in the cells was not limited only to these findings, and there was some evidence supporting the fact that they might have other roles in the cells. The results of molecular investigations shed light on the ability of TLRs in propagating specific signalling in the cells which regulates the balance between pro‐ and anti‐apoptotic target genes.^2^ Having established these regulatory functions, a new chapter has opened about the TLRs, introducing them as important regulators of tumorigenesis.

## 2 A GLIMPSE INTO THE BIOLOGY OF TLRs, FROM THEIR BIOGENESIS TO THE DEPTH OF THEIR FUNCTIONS

From the immunological point of view, the characteristics of these molecules have been well‐studied in different reports to unveil the structure, signalling and functions of TLRs in cells. The results of high‐resolution X‐ray crystallography analysis have revealed that TLRs deviate considerably from the canonical LRR structure and thereby they could interact with a wide range of ligands in a highly divergent fashion. This different structure gives TLRs the ability to be activated in a different manner from other LRRs, resulting in the involvement of these receptors in other biological processes rather than only regulating innate immunity.^3^ In the following part of this article, we take a look at the biogenesis of TLRs to become more aware of their unique biology. Then, a brief explanation would be provided about their structure, their probable ligands and downstream signalling pathways. As the propagation of the TLR cascade may result in the regulation of diverse intracellular functions, we dedicate the last part of this section to explain the biological functions of the molecules so that we can learn more deeply about the functions of TLRs.

### 2.1 A glance at TLR biogenesis and localization

All the TLRs are synthesized from their mRNAs into functional configurations in the endoplasmic reticulum (ER) and are translocated to the Golgi complex followed by trafficking to either the plasma membrane or endosomes. The localization of TLRs is a complicated process, which is controlled by a group of ER‐associated proteins. Thus far, ten functional TLRs have been identified in humans according to their subcellular localization. Although TLR1, TLR2, TLR4, TLR5, TLR6 and TLR10 are expressed on the cell surface and migrate to phagosomes after activation, the expression site of TLR3, TLR7, TLR8 and TLR9 is in intracellular compartments, in particular the endosomes and the endoplasmic reticulum. Among all, the subcellular localization of TLR4 is unique as this TLR could be found either at the plasma membrane or at endosomal vesicles.^4^

A member of the ER‐resident HSP90 protein family, gp96, serves as a general chaperone for the surface‐expressed TLR1, TLR2, TLR4 and TLR5, and intracellular TLR7 and TLR9. Deficiency in gp96 leads to the loss of expression of TLR1‐TLR5 and TLR7 or improper TLR9 protein folding.^5^ Unc‐93 homolog B1 (UNC93B1), a multi‐pass TM protein, controls the endosomal trafficking of TLRs, especially TLR3. For endosomal TLRs, such as TLR3, TLR7 and TLR9, a multi‐span transmembrane protein, UNC93B, enters the game to traffic these proteins to endosome.^4^ Another ER‐resident protein that regulates the trafficking of TLRs is named protein associated with TLR4 A (PRAT4 A), which on one hand guide TLR1, TLR2 and TLR4 to the cell membrane, and on the other hand propel TLR7 and TLR9 to endosomes.^6^ TLRs tend to construct dimers in the presence of ligand. Most of the TLRs appear to form homodimers; however, the story is quite different for TLR2 as it prefers to be presented as a heterodimer with either TLR1 or TLR6.

### 2.2 A glance at TLRs structure

TLRs belong to type I transmembrane glycoproteins and contain three major domains, ectodomain, single‐spanning transmembrane domain and cytoplasmic TLR domain. The ectodomain is oriented towards extracellular space or luminal space (depending on receptor localization) and contains multiple^7^, ^8^, ^9^, ^10^, ^11^, ^12^, ^13^, ^14^, ^15^, ^16^, ^17^, ^18^, ^19^ leucine‐rich repeats (LRRs) that harbour 24‐29 amino acids.^20^ Two types of motifs were considered for this domain: ‘typical’ (‘T’) motifs (LxxLxLxxNxLxxLxxxxF/LxxLxx) and ‘bacterial’ (‘S’) motifs (LxxLxLxxNx LxxLPx(x)LPxx).^20^ The unique horseshoe shape of the TLRs is established by folding LRR modules into parallel β‐sheets and α‐helix that bend into a concave surface.^21^ Moreover, as LRR hydrophobic residues are packed within the interior of the ectodomain structure and forming a ligand‐binding hydrophobic pocket, it is suggested that LRRs play a pivotal role in recognition and binding of pathogens. In addition, the C‐terminal of LRRs controls receptor dimerization and signal transmission. The next domain of TLRs is the single‐spanning transmembrane domain that is homologous to IL‐1R analogue and anchors the receptor in the correct orientation on the cell membrane. The third and the last domain in the structure of TLRs is the cytoplasmic TLR domain (Toll/interleukin‐1 receptor domain, TIR domain) that is usually composed of approximately 150 amino acid residues.^21^ Upon ligand‐ectodomain interaction and respective alterations in the receptor conformation, TIR domain dimerizes either in the form of hetero‐ or homodimers and initiates specific signalling pathway through engaging of a wide range of adaptor proteins that all contain TIR domain, including MyD88, TIRAP/MAL, TRIF, TRAM and SARM. All TLRs, except TLR3, utilize MyD88 for signal transduction upon ligand binding, whereas TLR3 signals through the adaptor TRIF. It should be noted that the dimerization of the TIR domain is necessary for the dimerization of the cytoplasmic domain.^22^

### 2.3 A glance at TLRs ligands

Although each TLR recognizes distinct ligands, the mechanisms of TLR activation and signal transduction are somehow the same. Several microbial ligands have been enumerated for TLRs, which their lists are summarized in Table 1. Microbial ligands are not the only stimulators of TLRs, and recently, a group of other proteins, referred to as alarmin, have been identified. They could propagate TLR signalling in the cells, especially through activating TLR2 and TLR4. The list of these endogenous ligands is also summarized in Table 1. According to the surveillance model proposed by Johnson et al, another sort of ligands that are responsible for TLR activation is known as the degradation products of endogenous macromolecules, such as heparan sulphate and polysaccharide fragments of hyaluronan.^23^ The signals that are mediated by the degenerated macromolecules mostly trigger TLRs‐mediated tissue repair processes in addition to initiating protective inflammatory responses.^24^

**TABLE 1:** List of ligands that could activate TLRs

	DAMPs	PAMPs	References
TLR1	β‐defensin 3	Lipoarabinomannan (mycobacteria), triacyl lipoproteins, peptidoglycan (Gram‐positive bacteria), zymosan (fungi)	85, 86
TLR2	Heat‐shock protein 60 (HSP60), HSP70, HSP96, high‐mobility group protein B1 (HMGB1), hyaluronic acid, human cardiac myosin and biglycan	Lipoprotein (bacteria), peptidoglycan (Gram‐positive bacteria), lipoteichoic acid (Gram‐positive bacteria), lipoarabinomannan (mycobacteria) and zymosan (fungi)	86, 87, 88
TLR3	mRNA	Viral dsRNA (WNV, RSV)	^88^
TLR4	HSP22, HSP60, HSP70, HMGB1, fibronectin, fibrinogen, hyaluronic acid, heparan sulphate and biglycan	LPS (Gram‐negative bacteria) and envelope proteins (MMTV and MMLV)	7, 8
TLR5		Flagellin (bacteria)	^87^
TLR6		Diacyl lipopeptides (mycoplasma), peptidoglycan (Gram‐positive bacteria) and zymosan (fungi)	87, 88
TLR7	Endogenous RNA	Viral single‐stranded RNA (ssRNA)	^9^
TLR8	Endogenous RNA	Viral single‐stranded RNA (ssRNA)	^9^
TLR9	Endogenous DNA	Unmethylated CpG motifs (bacteria and viruses) and haemozoin (plasmodium)	9, 10

### 2.4 TLR signalling

Given the importance and the key roles of TLRs in regulation of immune responses, it is not surprising that their emitted signals are a succession of complex events in the cells that lead to activation or suppression of a wide range of downstream signalling axes. Generally speaking, the TLR signalling pathway could be mediated through either MYD88‐dependent or MYD88‐independent manner. The TLR signalling has been well reviewed in an article provided by Luo et al^22^

### 2.5 TLR functions: from immunological perspective to non‐canonical functions

#### 2.5.1 Immunological functions of TLRs

##### Innate immune responses

When it comes to TLRs, the first thing that comes to mind is its bona fide action in response to specific molecules derived from bacteria and viruses.^24^ In the perspective of innate immunity, which TLRs endow their popularity from, these receptors play a fundamental role in phagocytosis of microorganisms and propagation of microbial killing through elevating the production of reactive oxygen and nitrogen intermediates. Moreover, the interaction of TLRs with their ligands could attract leucocytes to the infected organs either through regulating the surface expression of adhesion molecules or by inducing specific chemokines. Notably, TLRs also regulate the functions of potent antimicrobial factors, such as defensins (α and β), phospholipase A2, lysozyme and the regeneration (Reg) family of molecules.^11^

##### Adaptive immune responses

TLRs are indeed the main players of the innate immunity system; however, their central role in regulation of host protective adaptive immune responses should not be underestimated. It has been suggested that TLRs could stimulate both T cell‐ and B cell–mediated immune responses upon exposure to adjuvants containing microbial lysates or products.^11^ Moreover, TLRs could stimulate professional antigen‐presenting cells (APCs) and have a role in processing and presentation of microbial antigens, up‐regulation of co‐stimulatory molecules, T cell activation and suppression of regulatory T cells.^12^ TLR‐mediated production of IL‐12 dictates differentiation of activated T cells into T helper 1 (Th1) cells. TLRs are also crucial for activation and maturation of the B cell responses during infection and vaccination. Last but not least, through both T cell–dependent and T cell–independent pathways, TLRs regulate B cell proliferation, immunoglobulin isotype class switching and somatic hypermutation.^13^

#### 2.5.2 Non‐canonical functions of TLRs

**FIGURE 1:**
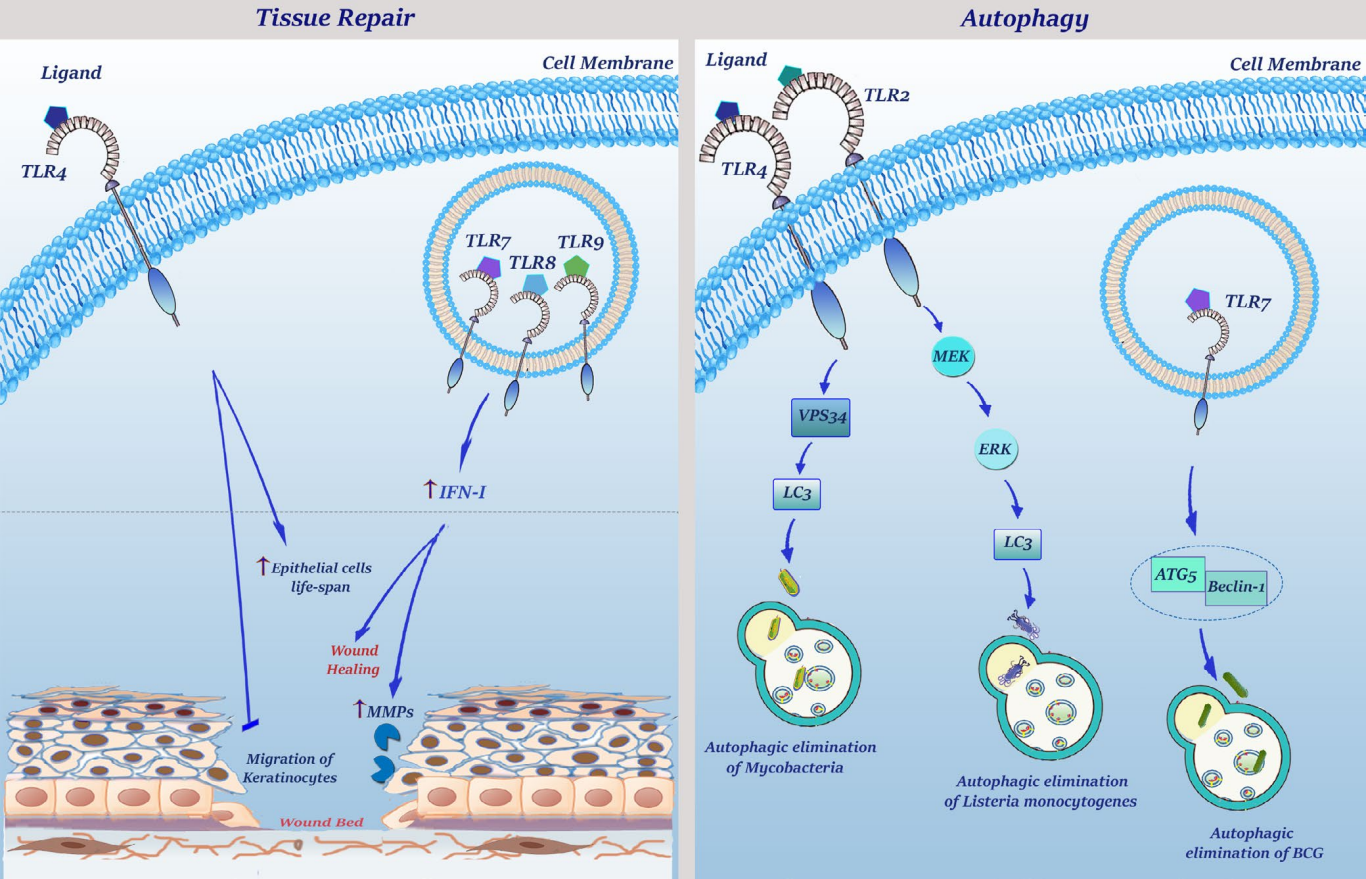
Non‐canonical functions of TLRs. TLRs play an important role in wound healing and tissue repair. TLR4 signalling inhibits migration of keratinocytes and its expression on epithelial cells results in an increased survival. Stimulation of TLR7, TLR8 and TLR9 have also anti‐apoptotic effects on fibroblasts through induction of type I interferons. In addition, TLRs can regulate wound healing response by attenuating fibroblast migration and increasing MMPs. Control and regulation of autophagy are another physiological function of TLRs. TLR4 signalling leads to LC3 aggregation via the TRIF signalling pathway which engages VPS34. Autophagic elimination of BCG can be triggered by TLR7 which uses the MyD88 signalling pathway to recruit Beclin‐1 and ATG5. TLR2 activation cascade also results in ERK phosphorylation, which in turn leads to the formation of the LC3 complex that is needed for *listeria monocytogenes* autophagic elimination

Apart from participation in the regulation of immune responses, which is well‐described in the previous reports, TLRs have some non‐canonical functions in tissue repair, regulation of apoptotic cell death and autophagy. Any aberrancy in the regulation of these receptors could orchestrate the initial step of tumour development, endangering normal human cells to form cancer cells (Figure 1).

##### Tissue repair

Several mechanisms have been enumerated for TLRs through which they could induce tissue repair and regeneration upon an injury. Generally, participation of TLRs in wound healing occurs at both early and delayed stages. Although at the early phase of wound healing TLRs recruit inflammatory cells to the injured site to produce cytoprotective signals through up‐regulating the expression of anti‐apoptotic genes, these receptors could modulate fibroblast recruitment and regenerative responses at the delayed phase. TLR4 could also increase epithelial cell life span and prevent the migration of keratinocytes.^14^ TLR3, as an endosomal TLR, participates in delayed wound healing that is mainly mediated through TRIF‐dependent type I‐IFN secretion. Of note, several studies also declared that TLRs can regulate extracellular matrix (ECM) metabolism by increasing the production of MMPs, such as MMP1, MMP3 and MMP9.^15^

##### Autophagy

When it became evident that activated TLR4 could enhance autophagic elimination of phagocytosed mycobacteria in macrophages via VPS34‐dependent formation of cytoplasmic LC3 aggregates, a new chapter has been opened in the biology of TLRs in the regulation of autophagy. It did not take long that the role of other TLRs in the regulation of autophagy has been established. TLR7 can eliminate bacillus Calmette‐Guerin (BCG) through up‐regulation of ATG5 and Beclin‐1.^16^ TLR2 eradicates *Listeria monocytogenes* through ERK‐mediated activation of autophagy. The more carefully the association of TLRs with the process of autophagy is studied, the more molecular mechanisms have been found to explain the precise role of this cascade in the activation of this self‐devouring event. It turns out that TLRs could recruit Beclin‐1, which in turn interacts with TRAF6, inducing K63‐polyubiquitination of the BH3 domain for its activation and subsequent formation of autophagosomes.^17^

## 3 TLRs IN CANCER: DOUBLE‐EDGED SWORDS

The first evidence supporting the involvement of TLRs in the tumorigenesis event has stemmed from a report indicated that the risk of cancer development is significantly higher in organs that are directly or indirectly exposed to bacterial TLR ligands. This finding is also reflected in the low incidence of cancer development in germ‐free animals.^18^ Although there remains much to learn about the involvement of TLRs in tumorigenesis, a question has occupied the mind of the researchers: How TLRs^___^that are responsible for the regulation of immune responses against unknown antigens, such as cancer cells^___^could play such a controversial mechanism of action? The answer to this question may be in the amplitude and length of receptor activation. Although chronic low‐grade TLR activation favours a tumour‐promoting pro‐inflammatory state, high‐dose TLR activation induces antitumour response.^19^

### 3.1 The pro‐tumour activity of TLRs

The idea behind the involvement of TLRs and their related signalling in the formation of human cancers has originated from the considerable number of studies reported the abnormal expression of TLRs on tumour cells, where they may influence tumour growth and immune responses.^25^ The discovery of these receptors in tumour cells has heralded a renaissance in the interconnection between innate immunity and tumour biology.

#### 3.1.1 Overexpression of TLRs in human cancers

Each TLR, either as a single‐player or as a team, could participate in the development of a specific type of tumour. When it comes to oral and gastrointestinal cancers, the first TLR that comes to mind is TLR2.^26^ TLR3 has shown to participate in the pathogenesis of neuroblastoma, breast adenocarcinoma, hepatocellular, papillary thyroid, nasopharyngeal and lung carcinomas.^27^ TLR4 is notorious for its fundamental participation in the pathogenesis of human lung cancer, neuroblastoma, colorectal cancer and thyroid carcinomas.^2^ Various levels of TLR9 expression have been demonstrated in tumour specimens from patients with prostate cancer, breast cancer, astrocytoma, lung cancer and glioblastoma.^28^ The involvement of TLR9 could be used as a risk stratification factor to categorize the cancer patient's prognosis and outcome; however, based on the type of cancer, it may be either attributed to the good or attributed to the poor prognosis. In renal cell carcinoma, mucoepidermoid salivary gland carcinoma and pancreatic cancer, the higher expression levels of TLR9 are indicative of a longer survival.^29^ However, the up‐regulation in TLR9 in oesophageal adenocarcinoma, squamous cell carcinoma of the tongue and prostate cancer is related to the more aggressive form of the disease and the dismal outcome.^30^

So far, we have looked at the association between the individual TLRs and the induction of human cancers; however, in most cases, these receptors act as a group to enhance the survival and proliferative capacity of the tumour cells. TLR4 and TLR9 could endow prostate cancer cells the ability to proliferate more aggressively.^31^ TLR9 and TLR5 could also make a team in the formation of cervical carcinogenesis, as there are some reports demonstrating that expressions of these TLRs are gradually increased during the progression of low‐grade cervical intraepithelial neoplasia (CIN) to high‐grade CIN and then to invasive cervical squamous cell carcinoma.^32^ Other examples of simultaneous expression of TLRs in cancer cells could be found in ovarian cancer cell lines which displayed overexpressed TLR2‐TLR5,^33^ and in lung cancer with overexpressed TLR2‐TLR4 and TLR9.^34^ Moreover, overexpression of TLR4, TLR5 and TLR9 not only has been reported in gastric cancer cell lines but also was observed in the metaplastic and dysplastic gastric epithelial cells of patients with *Helicobacter pylori* gastritis.^35^ One of the human cancers which displayed the overexpression of multiple TLRs is melanoma, in which up‐regulation of TLR2‐TLR4 is coupled with metastasis and tumour progression.^36^ The association between the overexpression of TLRs and human cancers is summarized in Table 2.

**TABLE 2:** The association between the overexpression of TLRs and the development of human cancers

	Up‐regulated TLRs	Outcome	References
Ovarian cancer	TLR4 TLR2‐TLR5	Recruits PI3K axis to increase XIAP leading to tumour growth and chemo‐resistance Inhibit apoptosis by increasing XIAP expression and Akt phosphorylation	^33^
Myeloma cells	TLR1, TLR7, TLR9	Prevent apoptosis through autocrine secretion of IL‐6 and induction of drug resistance	^37^
Breast cancer	TLR3, TLR4, TLR 9	Induce tumour metastasis through increasing of MMP13 and lipid peroxidation	^70^
Prostate cancer	TLR9	Induces metastasis through increasing MMP13 expression Enhancing cell survival and proliferation by activation of NF‐κB and c‐Myc	^55^
Melanoma	TLR2, TLR3, TLR4	Induce metastasis through increasing MMP13 Up‐regulation of pro‐inflammatory cytokines and chemokines, immunosuppressive cytokine	36, 56
Gastric cancer	TLR2, TLR9 TLR4, TLR5, TLR9 TLR2	Induce angiogenesis through elevation of COX‐2 and PGE2 expression Induce NF‐κB–dependent tumour invasion and metastasis Enhances tumour cells proliferation and survival through PI3K/Akt and NF‐κB axis	45, 60
Colorectal tumour	TLR4 TLR2	Induces angiogenesis through elevating COX‐2 and PGE2, as well as phosphorylation of EGFR Induces anti‐apoptotic effects through activation of PI3K/AKT and NF‐κB	^61^
HNSCC	TLR4 TLR3	Immune escape via secretion of IL‐6, IL‐8, VEGF and GM‐CSF and drug resistance Induces metabolic reprogramming by increasing HIF‐1α	69, 89
Colon cancer	TLR4	Apoptosis inhibition; relapse and metastasis	^90^
Pancreatic cancer	TLR2, TLR4, TLR9	Increase VEGF, PDGF, MAPK and pERK leading to inflammation and angiogenesis	^91^
Lung cancer	TLR2, TLR3, TLR4, TLR9 TLR4 TLR7, TLR8 TLR2	Resistance to TNF‐α or TRAIL‐induced apoptosis through NF‐κB up‐regulation Induces metastasis Increase the expression of Bcl‐2 by activating NF‐κB Increases tumour cells proliferation and IL‐8	^34^
Oral squamous cell carcinomas	TLR2 TLR3, TLR4 TLR4, TLR5	Up‐regulates the proliferative kinase ERK & reduce pro‐apoptotic caspase‐3 activity Induce metabolic reprogramming by increasing HIF‐1α Induce tumour invasion and metastasis	^92^
Bladder cancer	TLR2, TLR3, TLR4	Enhance transcription of genes involved in cell proliferation through activation of NK‐κB Induction of pro‐inflammatory and angiogenic factors such as COX‐2, VEGF and TGF‐β Conversion of M1 macrophage to M2	^93^
Hepatocellular carcinoma	TLR4 TLR2, TLR3, TLR6	Increases cell proliferation and survival through the NK‐κB and MAPK pathways Increases pro‐inflammatory factors like COX‐2 and prostaglandin through the STAT3 pathway Anti‐apoptotic effects by inhibition of caspase 3, 6, 7 and 9 transcription Increases tumour invasion and metastasis, especially macrovascular invasion Enhance cell proliferation and inflammation	94, 95
Oesophageal Cancer	TLR4 TLR3, TLR4, TLR7, TLR9	Immune escape and inflammation by up‐regulation of IL‐8 and COX‐2 Tumour metastasis via up‐regulation of p38 and selectin Enhance inflammation, invasion (especially lymph node metastasis) and proliferation	^96^
Adrenocortical carcinoma	TLR2, TLR4	Induction of inflammation via up‐regulation of TNF‐α, IL‐6 and IL‐8	^97^

Another critical complication that is associated with grouped TLRs expression is the regulation of drug‐resistance phenotype, which eventually lead to the poor outcome of patients and even increase the risk of cancer recurrence. Co‐expression of TLR1, TLR7 and TLR9 in myeloma cells has shown to be involved in induction of drug resistance.^37^ Moreover, the up‐regulation of TLR3, TLR4 and TLR9 expressions could increase the probability of biochemical recurrence and cancer metastasis in prostate and breast cancer, respectively.^38^ TLR4 and TLR9 could also orchestrate a signal that helps cancer cells to bypass the immune responses by increasing the expression of immunosuppressive cytokines and anti‐apoptosis proteins. It has been also indicated that the co‐stimulation of TLR7 and TLR8 could induce chemo‐resistance via NF‐κB–mediated up‐regulation of anti‐apoptotic members Bcl‐2 family in human lung cancer cells.^39^

#### 3.1.2 The mechanisms through which TLRs orchestrate pro‐tumour responses

**FIGURE 2:**
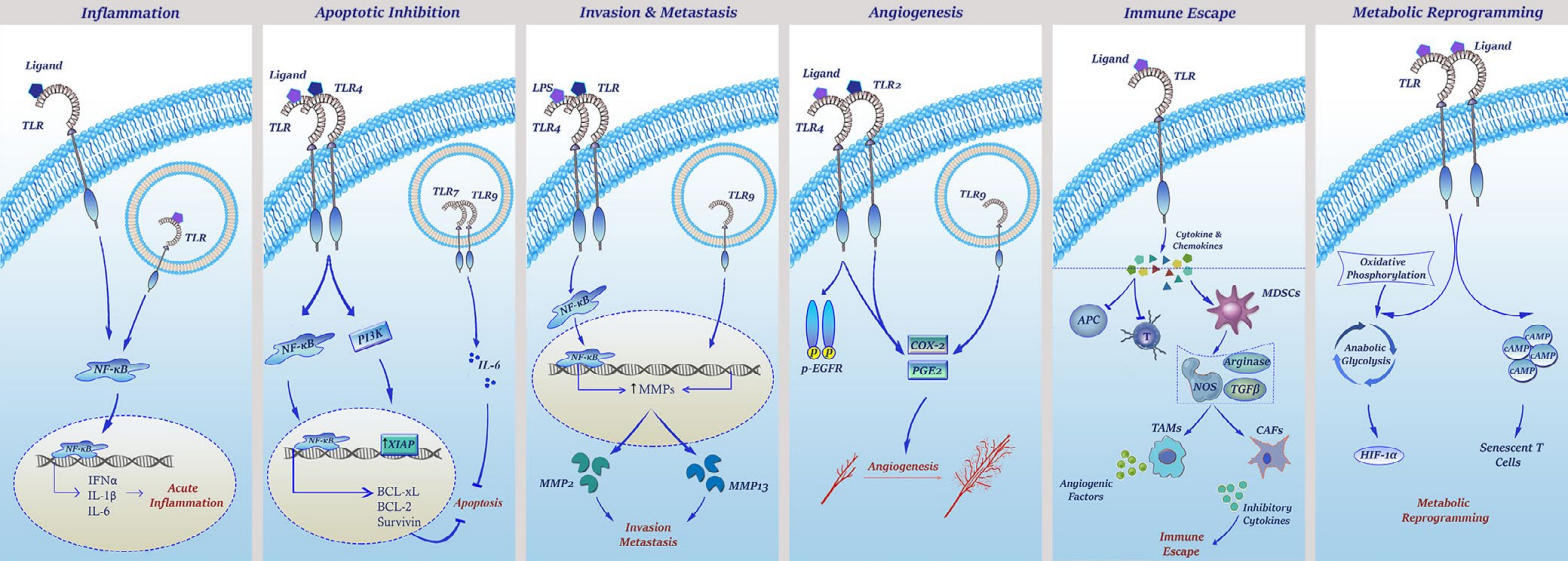
The mechanisms through which TLRs orchestrate pro‐tumour responses. TLR stimulation activates the NF‐κB cascade, which in turn increases the transcription of inflammatory cytokines including IFN‐α, IL‐1 and IL‐6. Activation of the NF‐κB axis not only promotes anti‐apoptotic factors (Bcl‐XL, Bcl‐2, survivin) but also up‐regulates MMPs which are responsible for the degradation of extracellular matrix. Although TLR4 activation leads to the production of MMP2 and β1‐integrin overexpression, TLR9 stimulation enhances MMP13 level in breast cancer. As represented, increasing of PG‐E2 and COX‐2 by TLR2, TLR4 and TLR9 and phosphorylation of EGFR by TLR4 are the most important mechanisms in TLR‐mediated angiogenesis. Stimulation of TLRs in the tumour microenvironment can provoke tumour cells or tumour‐infiltrating cells to produce suppressor cytokines and chemokines which not only suppress immune cells but also attract more cells to tumour microenvironment—such as MDSC, TAMs and CAFs—to fulfil tumour needs. Finally, TLR downstream signalling can make tumour cells and tumour‐infiltrating cells to switch their metabolic pathway from oxidative phosphorylation to glycolysis

The aberrant overexpression of TLRs on malignant cells and their well‐established association with the promotion of human cancers has put an end to the multiple questions wondering the role of TLRs in the regulation of tumorigenesis, especially at the early stage of the event. This causes the new stream of attempts to be accomplished for proposing the probable mechanisms through which TLRs could promote carcinogenesis (Figure 2).

##### TLR‐mediated inflammation

In 2000, Hanahan and Weinberg proposed a model to define ‘six hallmarks’ that a tumour requires for maintaining its survival.^40^ However, as the conventional knowledge about the pathogenesis of cancer was progressing, inflammation was also added as the seventh feature to the list.^41^ There are a considerable number of studies with the focus on the correlation between cancer development and the previous history of microbial infection, injury, inflammation and tissue repair. Although it is early to hazard a conjecture for the mechanism through which inflammation may develop a malignancy, the possible candidate could be TLRs that engage in a unique cross‐talk with the NF‐κB signalling axis. Through regulating more than a hundred pro‐inflammatory genes, NF‐κB is a master of inflammation regulator.^42^ When TLR signalling is stimulated, it recruits the NF‐κB signalling axis to increase the expression of inflammatory cytokines, such as interleukin (IL)‐1β, tumour necrosis factor α (TNF‐α) and IL‐6.^42^ These cytokines are notorious for their role in the induction of cancer in the intestine, liver, stomach and skin.^43^ Apart from regulation of pro‐inflammatory cytokines, TLR‐mediated activation of the NF‐κB signalling axis also regulates a wide variety of intracellular responses, including cell proliferation, anti‐oxidant defence and prevention of apoptosis in the malignant cells.^44^ The association between TLRs and inflammatory responses, indeed, makes a vicious cycle; the chronic inflammation induces oxidative stress, leading to the formation of oxidized lipids, which by their deformed patterns act as DAMPs to re‐activate a group of TLRs.

##### TLR‐mediated anti‐apoptotic effects

As mentioned earlier, the TLR network has a tight cross‐talk with the NF‐κB axis through either MYD88‐dependent or MYD8‐independent manner.^44^ As such, TLR‐induced NF‐κB activation inhibits apoptosis and promotes tumour cell survival in colon cancer, liver cancer, stomach cancer and lung cancer.^39^, ^45^ Independent of the NF‐κB axis, it has been claimed that the excessive expression of TLR4 on tumour cells blocks the cytotoxic effects of T lymphocytes and enhances the growth of the tumour in vivo.^46^ In a study in ovarian cancer cells, Kelly et al showed that the activation of TLR4 signalling promotes tumour growth and induces chemo‐resistance through recruiting the PI3K signalling axis, which in turn increases the expression of X‐linked inhibitor of apoptosis (XIAP) in the malignant cells.^47^ The oncogenic effect has also been reported for TLR7 and TLR9 in the myeloma cells, where the aberrant expression of such receptors prevents chemotherapy‐induced apoptosis through promoting the autocrine secretion of IL‐6.^37^, ^48^ Besides, it has been revealed that the inhibition of TLR4 using small molecule inhibitor TAK‐242 suppressed NF‐кB–related anti‐apoptosis genes BCL‐xL, BCL‐2 and survivin in breast and ovarian cancer cells and led to increased apoptosis.^49^, ^50^

In a recent study, we also examined the combination of TAK‐242 and four well‐known chemotherapeutic agents: paclitaxel, cisplatin, doxorubicin and arsenic trioxide in breast and ovarian cancer cells. Interestingly, we realized that the inhibition of TLR4 boosted the cell cytotoxicity of all drugs, which indicates the fact TLR4 would confer chemo‐resistance to a broad range of anti‐cancer agents.^51^, ^52^

##### TLR‐mediated invasion and metastasis

Having established the role of TLR‐associated signalling pathways in multiple steps of tumorigenesis, intense interest has been attracted to investigate whether this network could also participate in tumour cell invasion and metastasis. In similarity with other stages in tumorigenesis, the results of two studies showed that TLR activation in tumour cells increases the tendency of tumour cells to adhere to the extracellular matrix and endothelial cells, which ultimately elevates the risk of tumour metastasis. It has been shown that LPS promotes tumour invasion through the TLR4‐mediated activation of the NF‐κB pathway, resulting in the up‐regulation of matrix metalloproteinase 2 (MMP2) and the β1‐integrin subunit.^53^ Merrell et al also showed that stimulation of TLR9‐expressing breast cancer cells with CpG ODNs dramatically increased their in vitro invasion by increasing the activity of MMP13.^54^ Another in vitro study suggested that TLR9 agonists can stimulate prostate cancer invasion by increasing MMP13 activity.^55^ Moreover, the interaction of TLR2, TLR3 and TLR4 with their ligands on human melanoma cells was associated with increased cell migration and tumour metastasis.^56^ Tumour‐secreted miR‐21 and miR‐29 also act as paracrine agonists of TLRs, which through interacting with either murine TLR7 or human TLR8 on immune cells transmit signals between tumour cells and the microenvironment, leading to regulation of tumour metastasis.^57^, ^58^ The results of our recent studies revealed that TLR4 blockade using highly selective TLR4 inhibitor TAK‐242 suppresses ovarian and breast cancer cell invasion through the inhibition of EMT. In this study, we showed that not only did TAK‐242 reduce the enzymatic activity of MMP2 and MMP9 but also down‐regulated the mRNA expressions of genes involved in both ECM degradation and EMT‐related genes including uPA, uPAR, ZEB1, SNAIL1, SNAIL2 (SLUG), CDH2 and β‐catenin.^59^

##### TLR‐mediated angiogenesis

Since vascular endothelial growth factor (VEGF), the main factor involved in tumour angiogenesis can be induced by activation of TLRs, and it is not surprising to bring up TLRs as potent regulators of tumour angiogenesis. The first study that introduced the role of TLRs in tumour angiogenesis was conducted on *H .pylori*–associated gastric cancer, in which it has shown that *H .pylori*–induced cyclooxygenase‐2 (COX‐2) and prostaglandin E2 (PGE2) expression enhanced tumour angiogenesis via interacting with TLR2 and TLR9.^60^ Another in vitro study found a direct endothelial stimulatory role for LPS in initiating angiogenesis through activation of TLR signalling pathways. It became evident that TLR4 promotes colitis‐associated colorectal tumours through phosphorylating epidermal growth factor receptor (EGFR) and inducing COX‐2 and PGE2.^61^ In another study, TLR4 deficiency protects mice from colitis‐associated neoplasia because of the decreased level of mucosal PGE2.^62^ Taken together, these findings are highlighting the importance of TLR‐associated signals in the regulation of tumour angiogenesis, which not only increase the metabolic activity of malignant cells but also elevate the risk of tumour invasion to other organs.

##### TLR‐mediated immune escape

Although TLRs are the main player in the regulation of immune responses, their aberrant expressions on tumour cells could confer the resistant phenotype to malignant cells against cytotoxic effects of lymphocytes through production of some pro‐inflammatory compounds. During cancer progression in the setting of chronic inflammation, integration of TLRs on the tumour cells or tumour‐infiltrating immune cells and their ligands leads to the secretion of cytokines and chemokines from these cells into the tumour microenvironment. The released cytokines basically suppress the immune response, either by impairing the function of APCs, T cells and TAA‐specific immunity or by recruiting more immune suppressive cells to the tumour nidus, leading to the production of more inflammatory cytokines that activate cancer‐associated fibroblasts (CAFs).^63^ The suppressive cytokines, such as IL‐10, IL‐6, IL‐8, VEGF and granulocyte‐macrophage colony‐stimulating factor (GM‐CSF) ease immune escape.^64^ IL‐10 also induces tumour‐associated macrophages (TAMs), which in turn release angiogenic and lymphangiogenic factors that promote lymphatic metastasis of cancer cells.^65^ The inflammatory cytokines, such as IL‐1b, IL‐6 and PGE2 recruit myeloid‐derived suppressor cells (MDSCs) into the tumour microenvironment, which aid cancer progression through releasing arginase, nitric oxide synthase (NOS) and TGF‐β.^66^ The secreted TGF‐β activates CAFs, which promote the proliferation and progression of cancer through the production of growth factors and metalloproteinases.^67^

##### TLR‐mediated metabolic reprogramming in the tumour microenvironment

Malignant tumour cells selectively reprogramme their metabolism to meet the rapid energy requirements for proliferation, survival and metastasis as well as for sustaining the tumour‐suppressive microenvironment.^68^ Tumour cells are not the only cells that benefit from metabolic reprogramming, as the other members of the tumour microenvironment including DCs, macrophages and T cells have shown to have hypoxic and acidotic conditions.^68^ More recent studies suggest that TLRs may directly regulate cell metabolism and thereby have an effect on tumour behaviours and functions. TLR3 promotes metabolic reprogramming of head and neck carcinoma cells and increases tumour growth and proliferation. It has been suggested that TLR3 can force tumour cells to switch from oxidative phosphorylation (OXPHOS) to glycolysis, which subsequently increases the expression of the transcription factor HIF‐1α and regulates hypoxia.^69^ In similarity to TLR3, there are also some studies reporting the importance of TLR9 in the regulation of lipid peroxidation in patients with breast carcinoma.^70^ In addition to directly affecting tumour metabolic reprogramming, TLRs can regulate cancer cell metabolites and indirectly influence antitumour immune responses in the tumour microenvironment. Through TLR‐dependent production of endogenous cAMP, human tumour cells can convert naive/effector T cells into senescent T cells to induce immune tolerance.^71^

### 3.2 The anti‐tumour activity of TLRs

**FIGURE 3:**
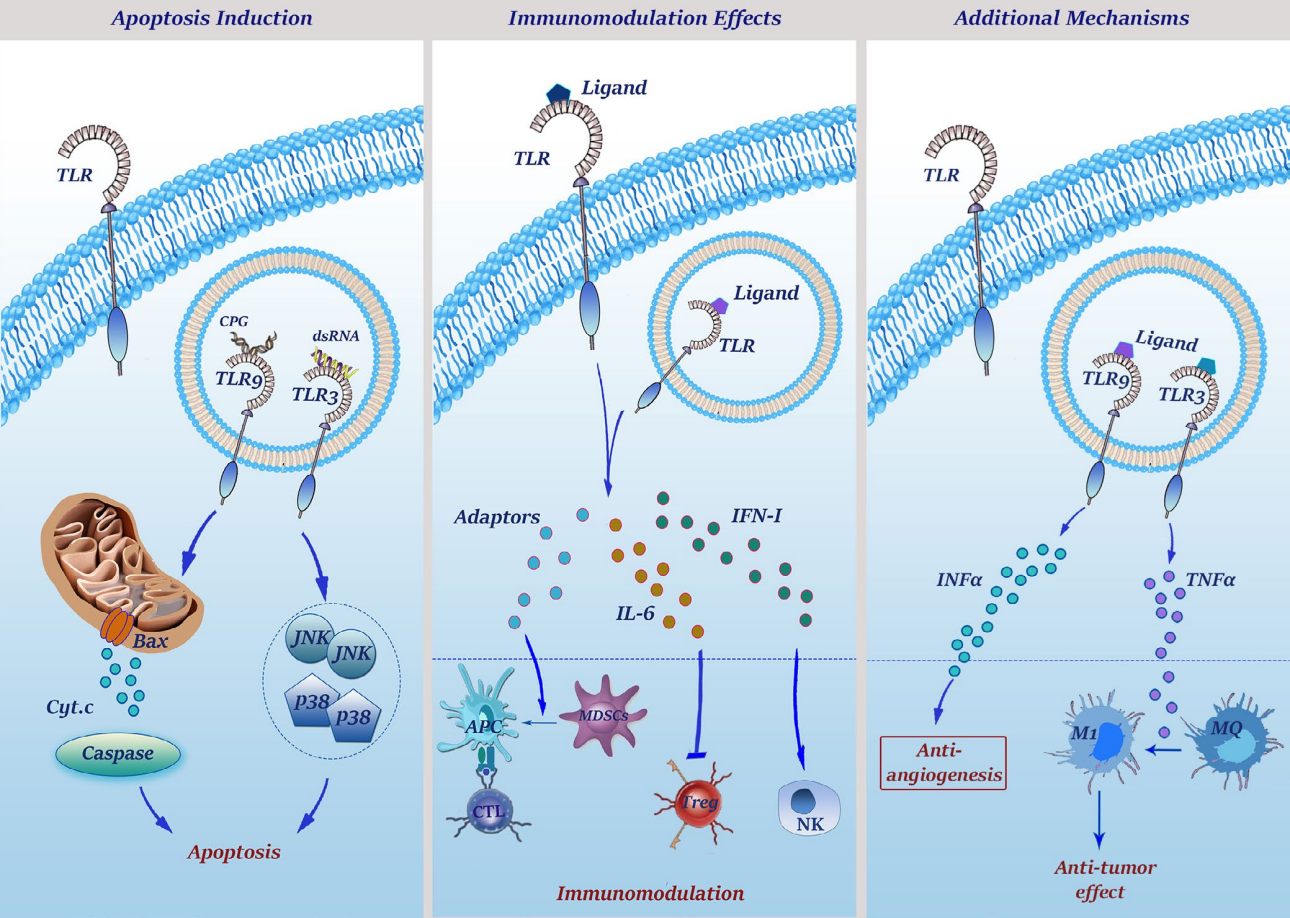
The mechanisms through which TLRs orchestrate anti‐tumour responses. TLR3 signalling can up‐regulate JNK and p38 which play important roles in regulation and induction of apoptosis. Moreover, TLR3 stimulation can take the control of p53 transcription and caspase cascade through up‐regulation of IFN‐γ. TLR9 activation, on the other hand, up‐regulates Bax which inserts into mitochondria membrane and makes it permeable to cytochrome C that is capable to initiate caspase‐dependent apoptosis. Because of the high‐level expression of TLRs on DCs, TLR agonists can make these cells present TAAs to T cells optimally. Activated DCs, in turn, could activate NK cells by secretion of type I IFNs and using INAM‐INAM interaction. On the other hand, activation of MDSCs by TLR7, 9 agonists inhibit their suppressor effects, leading to their maturation into APCs with the ability to elicit T cell anti‐tumour response. Moreover, stimulation of TLRs not only stops inhibitory functions of Tregs via IL‐6 induction but also suppresses their recruitment to the tumour microenvironment. Finally, stimulation of TLR3 with poly I:C up‐regulates TNF‐α that induces M1 macrophage phenotype with an important role in tumour regression

The complexities of TLRs and their associated signalling pathways have portrayed another picture for this cascade as a defender against human cancers. It has been suggested that the activation of TLRs in the cells could allay immune responses to trigger an anti‐cancer signal (Figure 3).

#### 3.2.1 A history focusing on anti‐tumour activity of TLRs

Evidence of antitumour effects of microbial products can be dated back to the early 18^th^ century when Deidier (1725) reported that infection in cancer patients could be concomitant with the remission of malignant diseases. In the 1890s, William B. Coley observed that repeated injections of a mixture of bacterial toxins were effective for the treatment of cancer patients. Although Coley could not explain the mechanism through which the bacteria induced anti‐tumour activity, the results of this observation introduced bacterial toxins as an effective therapeutic strategy against human cancers. In 1943, Shear and Turner discovered that LPS had anti‐cancer properties.^72^ Coley's notion of the antitumour activity of bacterial extracts has built a foundation of the theory that bacterial components, such as bacterial endo/exotoxins, lipoteichoic acid and bacterial DNA have strong antitumour activities through either inducing tumoricidal effects or enhancing the activation of the innate immune system.^73^ This idea was then revisited and explored by many researchers, leading to an understanding that microbe‐derived therapeutics may recruit TLR signalling to exert anti‐tumour activity through stimulating both innate and adaptive immune responses. TLR‐associated signalling and the activation of the downstream mediators, such as type I IFNs, could be therapeutically used to shift the balance from immunotolerance to antitumour effects. TLR7 agonists and IFN‐alpha have also shown promising therapeutic results in melanoma, basal cell cancer, renal cell cancer and hairy cell leukaemia.^74^ These findings aroused a stream of studies concentrating on the anti‐tumour activities for TLRs.

#### 3.2.2 The mechanisms through which TLRs orchestrate anti‐tumour responses

The results of deep molecular investigations proposed that TLRs could exert their anti‐tumour activity either by regulation of the apoptotic pathway or through modulating the activity of the immune cells, such as DCs, NK cells and T lymphocytes.

##### The ability of TLRs in induction of apoptotic cell death in cancer cells

Although multiple lines of evidence emphasized the anti‐apoptotic effects of TLRs, in many cases, there are conflicting results. TLR9 is the best example of this controversy as the apoptosis‐inducing capacity of this receptor is mediated through the mitochondrial‐dependent pathway. It has been shown that CpG‐DNA–mediated activation of TLR9 not only increased the expression of Bax but also induced caspase‐dependent apoptotic cell death in murine macrophages.^75^ Moreover, the interaction of TLR3 with dsRNA triggers a cascade to activate a wide range of downstream axes, such as p38, JNK and IFN regulatory factors, which in turn regulate apoptosis in cancer cells.^76^ TLR3‐poly I:C interaction also enhances the therapeutic value of the conventional antitumour agent cycloheximide in different human and murine tumour cell lines through increasing the production of IFN‐γ, a well‐known cytokine which its association with caspase and p53 has been previously well‐established.^77^

##### Immunomodulation through TLR signalling

Through inducing the cytotoxic signalling, mobilizing NK and T cells, and producing the anti‐tumour antibodies, the immune system could conveniently take hold of cancer cells. Being the most important components of the immune system and being expressed in most, if not all, immune cells, it is not surprising that TLRs may play a part in the anti‐cancer effects of the immune system. As professional APCs, which express a large number of TLRs, DCs are at the interface of innate and adaptive immune responses. Activated DCs amplify immune system‐induced anti‐tumour responses through presenting tumour antigens to cytotoxic T lymphocytes. The activation of DCs could be mediated through several mechanisms, but one of the most important ones is the stimulation of the TLR signalling pathway. TLR5, TLR7 and TLR9 are the best examples of TLRs that could reinforce the anti‐tumour activity of DCs.^78^ Not only TLRs could convert mature MDSCs to professional APCs and facilitate the presentation of tumour antigens to the cytotoxic T cells but also could suppress the activity of Tregs through secretion of IL‐6.^79^ TLR‐activated DCs could also make a partnership with NK cells through INAM interaction and secreting type I IFNs to activate them that leads to eradication of malignant cells.^80^ It has been reported that TLR3 stimulation in a mouse model of melanoma repressed the progression of tumour through myeloid DC‐mediated activation of NK cells.^81^ It should be noted that all TLRs do not exert their anti‐tumour activities through regulation of DCs. For example, TLR8 could either directly suppress the activity of Tregs or reduce the recruitment of Tregs to the tumour cells through secreting CCL22.^82^

##### Additional mechanisms through which TLRs exert antitumour effects

Converting tumour‐supporting macrophages to tumour suppressors that produce inflammatory cytokines and promote M1 polarization is another suggested mechanism that has been attributed to the anti‐tumour activity of poly I:C‐activated TLR3. This response is mediated by TNF‐α through a MyD88‐independent pathway.^83^ TLR9 agonists can also exert antitumour effects through suppression of angiogenesis. It is likely that the production of IFNs, such as IFN‐alpha, also plays a key role in both anti‐angiogenetic and tumour‐suppressive effects of TLRs.^84^

## 4 CONCLUSION AND FUTURE PERSPECTIVE

In many cases, cancer cells mimic many characteristics of immune cells. So, under the mask of these cells, neoplastic cells communicate and modulate the immune system for their own survival and growth. Despite several reports shedding light on the association between TLRs and the incidence of tumorigenesis, a considerable number of questions remained unanswered about the precise mechanisms of these receptors in cancer development. Given this, further exploration and more precise understanding concerning the role of TLRs in the maintenance of cancer cells are required to increase the current knowledge about their participation in tumour biology. These findings could also shed more light on the molecular basis of innate immunity, tumorigenesis and cancer biology, which all together could start a new chapter in the modern understating of cancer and its treatment strategies.

## CONFLICT OF INTEREST

The authors declare that they have no conflict of interest.

## AUTHOR CONTRIBUTIONS

**Yazdan Mokhtari :** Conceptualization (equal); Data curation (equal); Writing‐original draft (equal); Writing‐review & editing (equal). **Atieh Pourbagheri‐Sigaroodi :** Conceptualization (equal); Data curation (equal); Visualization (equal); Writing‐original draft (equal). **Parisa Zafari :** Conceptualization (supporting); Data curation (equal); Validation (equal). **Nader Bagheri :** Conceptualization (supporting); Validation (equal); Writing‐review & editing (equal). **Seyed H Ghaffari:** Conceptualization (supporting); Supervision (equal); Writing‐review & editing (equal). **Davood Bashash:** Conceptualization (lead); Project administration (lead); Supervision (lead); Validation (lead); Writing‐review & editing (lead).

## Data Availability

Data sharing is not applicable to this article as no data sets were generated or analysed during the current study.
